# Detecting heart failure from B-mode ultrasound characterization of arterial pulse waves

**DOI:** 10.1152/ajpheart.00219.2024

**Published:** 2024-05-24

**Authors:** Ryan M. Reavette, Anenta Ramakrishnan, Ethan M. Rowland, Meng-Xing Tang, Jamil Mayet, Peter D. Weinberg

**Affiliations:** ^1^Department of Bioengineering, Imperial College, London, United Kingdom; ^2^Department of Cardiology, The Hammersmith Hospital, Imperial College Healthcare NHS Trust, London, United Kingdom

**Keywords:** cross correlation, high-frame-rate ultrasound, systolic time intervals, ultrasound image velocimetry, wave intensity analysis

## Abstract

This study investigated the sensitivity and specificity of identifying heart failure with reduced ejection fraction (HFrEF) from measurements of the intensity and timing of arterial pulse waves. Previously validated methods combining ultrafast B-mode ultrasound, plane-wave transmission, singular value decomposition (SVD), and speckle tracking were used to characterize the compression and decompression (“S” and “D”) waves occurring in early and late systole, respectively, in the carotid arteries of outpatients with left ventricular ejection fraction (LVEF) < 40%, determined by echocardiography, and signs and symptoms of heart failure, or with LVEF ≥ 50% and no signs or symptoms of heart failure. On average, the HFrEF group had significantly reduced S-wave intensity and energy, a greater interval between the R wave of the ECG and the S wave, a reduced interval between the S and D waves, and an increase in the S-wave shift (SWS), a novel metric that characterizes the shift in timing of the S wave away from the R wave of the ECG and toward the D wave (all *P* < 0.01). Receiver operating characteristics (ROCs) were used to quantify for the first time how well wave metrics classified individual participants. S-wave intensity and energy gave areas under the ROC of 0.76–0.83, the ECG-S-wave interval gave 0.85–0.88, and the S-wave shift gave 0.88–0.92. Hence the methods, which are simple to use and do not require complex interpretation, provide sensitive and specific identification of HFrEF. If similar results were obtained in primary care, they could form the basis of techniques for heart failure screening.

**NEW & NOTEWORTHY** We show that heart failure with reduced ejection fraction can be detected with excellent sensitivity and specificity in individual patients by using B-mode ultrasound to detect altered pulse wave intensity and timing in the carotid artery.

## INTRODUCTION

Aortic root blood pressure, blood velocity, and diameter increase at the start of ventricular ejection. All three properties decrease when contraction diminishes later in systole. These two sets of disturbances propagate through the systemic arteries as waves. The wave at the start of ventricular ejection is a forward compression wave; that is, it travels from the heart toward the periphery and accelerates the blood. The wave that occurs when contraction slows is a forward decompression wave (also termed an expansion wave); it, too, travels from the heart to the periphery but decelerates the blood. Both waves are reflected from points where the anatomy or mechanical properties of the vessels change [see Ref. (1) for a review of pulse wave mechanics]. The two forward-traveling waves have been designated “S” (or “W1”) and “D” (or “W2”), respectively. The reflection of the S wave has been designated the “R wave.”

Curtis et al. (2) found that the energy in the carotid S wave was reduced by ∼40% in stable compensated systolic heart failure. There was also an increased period between the R wave of the ECG and the S wave and a decrease in the period between the S and D waves. The D-wave intensity was unaffected. Sugawara et al. (3) similarly found that the intensity of the S wave in the carotid was reduced by ∼50% in patients with dilated cardiomyopathy, without significant change in the D wave. They further showed that the intensity of the D wave was reduced by 50% in patients with hypertrophic cardiomyopathy, without significant change in the S wave. Hence wave intensity and timing may be of use in identifying heart failure and its subtypes.

Several technical hurdles need addressing before wave properties can be assessed routinely in clinical practice. In the original formulation of Parker and Jones (4), wave intensity, d*I*, is given by:

(*1*) dI=dPdU

where d*P* and d*U* are the changes in blood pressure (P) and velocity (*U*), respectively, over a short time interval in the artery of interest. [Wave energy is the integral of d*I* over time (1), and wave power is given by dP/d*Q*, where *Q* is the volumetric flow rate (5).] Ideally, P and *U* should be measured simultaneously, at the same arterial site, and with high temporal resolution. That is challenging: apart from measurement by intra-arterial catheter, which is not practicable for most patients, techniques for measuring P are cumbersome, inaccurate, or cannot be used at the time and site used to measure *U*.

Feng and Khir (6) developed an alternative definition of wave intensity:

(*2*) dIn=dDdU

where d*D* is the change in vessel diameter. Biglino et al. (7) proposed a formulation based on Q and cross-sectional area (*A*). The wave intensities obtained by these methods are not the same as those derived from dP and d*U*, but the methods are internally consistent and should have equivalent clinical utility (8, 9). Their advantage, symbolized by the prefix “*n*” in *Eq. 2*, is that *U* and *D*, or Q and *A*, can be obtained noninvasively. This has been achieved by using Doppler ultrasound to obtain *U* and B-mode or M-mode ultrasound to obtain *D* (10–12), or by using MRI to obtain *U* and *D* or *Q* and *A* (7, 13, 14).

We have argued (15) that the accuracy or utility of these methods suffers from well-established biases in Doppler velocity measurements (16), the 90° difference in optimal beam angle for Doppler and B-mode or M-mode imaging, and the high cost and long acquisition time of MRI. We showed that *D* and *U* can instead be measured in the same sequence of B-mode ultrasound images by using cross-correlation techniques to track speckles in the moving wall and blood (15). Blood speckles, which are normally obscured by the stronger clutter and wall signals, were detected after spatiotemporal filtering that used singular value decomposition (SVD), and adequate temporal resolution was obtained by acquiring images with an ultrafast scanner employing plane-wave transmission. The methods were successfully tested in the rabbit aorta, where they agreed with “gold standard” invasive methods and could detect pharmacologically induced ventricular dysfunction, and in a healthy human participant.

Here we report a feasibility study in which the methods were applied to participants having heart failure with reduced ejection fraction (HFrEF) and to control participants. We demonstrate for the first time the high sensitivity and specificity of various wave properties in identifying HFrEF, suggesting that the methods have the potential for screening and monitoring individual patients.

## METHODS

### Recruitment

Participants were recruited across community and hospital-based sites of the Imperial College Healthcare NHS Trust, which serves an ethnically and socioeconomically diverse population in North-West London. They were invited to participate in the study on attending an outpatient investigation. Approximately, a third of participants were recruited from the hypertension clinic or were undergoing blood pressure investigations, a third from the heart failure clinic, and a third following a referral for a transthoracic echocardiogram for various reasons.

For inclusion in the heart failure group, participants had to meet the European Society of Cardiology criteria for HFrEF according to a cardiologist: signs and/or symptoms of heart failure and left ventricular ejection fraction (LVEF) <40% (17). Participants included in the control group did not have signs or symptoms of heart failure and in addition had LVEF ≥50%. Patients showing clinical signs or symptoms of hypervolemia, as assessed by a cardiologist, were excluded. Participants under the age of 40 were also excluded to prevent overrepresentation in the control group.

All transthoracic echocardiography was performed or supervised and analyzed by sonographers with British Society of Echocardiography (BSE) accreditation and in accordance with BSE guidance (18). Wave data were collected on the same day as echocardiography where possible, but due to the COVID-19 pandemic, that was not always feasible. In those instances, the transthoracic echocardiography performed nearest to the date of recruitment was used. Participants without an echocardiogram performed within 12 mo of recruitment were excluded from the analysis. The interval for the remaining participants averaged 72 days but was over >180 days in 10 of them, including 3 with HFrEF. Exploratory analysis of S-wave intensity in the right carotid showed that excluding participants with delays >180 days had little effect (data not shown).

The study was approved by the National Health Service (IRAS ID 248724, HRA REC reference 18/SC/0563) and the Imperial College Healthcare NHS Trust and was registered as a clinical trial (ISRCTN 41232). It conformed with the Declaration of Helsinki. Participants gave written informed consent.

### Image Acquisition

At the time of scanning, the operator was blinded to the estimated LVEF of the participant. Participants were positioned semireclined at 45–60°. Left and/or right common carotid arteries were scanned in all participants, with both vessels scanned in the majority. ECG recordings with an identifiable R wave after bandpass filtering at 1–25 Hz were synchronized with the ultrasound scans in 72% of participants. Brachial blood pressure was measured using validated automated oscillometric devices. Participants were in the room for an average of ≈30 min.

To image the carotid, the participant’s head was tilted 45° away from the artery with the neck relaxed and the operator in the lateral position. Views were obtained proximal to the carotid bifurcation. Transverse views were taken to confirm the location of the vessel, and longitudinal views were obtained and recorded thereafter. Triplicate scans were normally obtained for each artery of each participant, with only one being selected for final analysis.

Scans were acquired with a research ultrasound system (Verasonics Vantage 64; Verasonics, Kirkland, WA) equipped with a 64-element L11-4V transducer. B-mode imaging was performed using a coherent plane-wave compounding scheme (19). Groups of three plane waves at −5°, 0°, and 5° were repeated to give a frame rate of 750–1,000 FPS. Each scan lasted 6 s, sufficient to capture a normal breathing cycle. Frequencies and voltages were 5.5–10 MHz and 30–75 V, respectively, giving mechanical indices in the range of 0.3–0.8, which is well within clinical guidelines.

Processing was performed by nonclinicians, blinded to the classification and echocardiography data. The methods involved only minor modifications of our previous techniques and are described in outline; see the studies by Rowland et al. (15), Reavette (20), and Riemer et al. (21) for further details.

### Preprocessing

Radiofrequency data were beamformed using an in-house delay-and-sum beamformer. Videos were filtered using SVD (15, 22).

### Diameter Tracking

Two rectangular regions, one on the top and one on the bottom wall, were selected in the first image of a video sequence (Supplemental Fig. S1*A*).

The region in the second image having the maximum correlation with the top region in the first image was identified as its new location. The same procedure was conducted for the bottom region. This process was repeated using the identified regions in the second image as the new starting point and calculating cross correlations with the third image, and so on. Regions were tracked throughout the video, giving two traces, one for the top wall and one for the bottom wall (Supplemental Fig. S1*B*). The distance between these traces gives the diameter trace (Supplemental Fig. S1*C*).

### Ultrasound Image Velocimetry

A rectangular region of blood speckle (Supplemental Fig. S2*A*) was tracked between successive frames using essentially the same cross-correlation method; its velocity was calculated from its displacement and the frame rate. For scans in which the arterial axis was slanted relative to the video frame axis, two-dimensional cross correlation was used to determine the separate *x*- and *z*-velocity components, and the axial blood velocity was found by combining them. Velocity traces were typically jagged (Supplemental Fig. S2*B*) and were therefore smoothed with a Savitzky–Golay filter (Supplemental Fig. S2*C*).

In a modification of our procedure for the aorta, the velocity was assumed to be constant across the artery (i.e., the velocity profile was blunt). As shown previously (15), this might have affected absolute wave intensities and energies, but not the relative differences between HFrEF and control groups.

### Wave Intensity Analysis

Noninvasive wave intensity was calculated using *Eq. 3*, a modification of *Eq. 2* that includes scaling of d*U* and d*D* by the timestep, Δ*t*:

(*3*) dIn=dDdU/(Δt)2

This removed the dependence of the magnitudes on the frame rate (23), which varied between acquisitions. Wave intensity waveforms were Savitzky–Golay filtered and then ensemble averaged (Supplemental Fig. S3).

### Wave Metrics

The basic metrics were the peak intensity of the S, R, and D waves (SWI, RWI, and DWI, respectively); the energy of the S, R, and D waves (SWE, RWE, and DWE, respectively); the S-D and ECG-S intervals, defined respectively as the time between the arrival of the peaks of the S and D waves and between the peak of the R wave of the ECG and the peak of the arterial S wave; and the reflection index, defined as RWI/SWI (see Supplemental Fig. S4 for a diagrammatic representation).

A new metric, termed the S-wave shift (SWS), was calculated as:

(*4*) SWS=(60×ECG-S interval)/(S-D interval×HR)

where HR is the heart rate (in beats/min) and the constant gives units of seconds.

### Statistics

Differences in characteristics between heart failure and control participants were assessed by the Student’s unpaired *t* test or, for the male:female ratio, by the χ^2^ test. Analysis of covariance tests was used to compare wave metrics between the two groups, with age as a covariate. Test-retest reliability was assessed by intraclass correlation, which compares the variability of repeat measurements in the same participant to the total variation across all measurements and participants (24). Confidence intervals for receiver operating characteristics (ROCs) were generated by bootstrapping.

## RESULTS

### Recruitment

Participant characteristics for the heart failure and control groups are shown in Table 1. There were no significant differences between the groups in age, male:female ratio, blood pressure, or HR. Significant differences occurred in LVEF and left ventricular end-diastolic diameter, but note that LVEF was used to define the two groups. The difference in *E/e′* was of borderline significance.

**Table 1.** Characteristics of control and heart failure groups

	Control	Heart Failure	*P* Value
*n*	38	19	
Age, yr	64 (12)	68 (12)	0.22
Male, %	47	63	0.26
SBP, mmHg	134 (16)	124 (20)	0.06
DBP, mmHg	79 (11)	77 (15)	0.48
HR, beats/min	69 (12)	70 (14)	0.78
LVEF	60 (3.7, 31)	31 (7.7, 18)	<0.001
*E/e′*	9.5 (3.9, 34)	13 (6.8, 4)	0.051
LVEDD, cm	4.5 (0.7, 37)	5.4 (0.8, 19)	<0.001

Values are means (SD); *n* = 38 control and 19 participants with HFrEF, except for the echocardiographic indices where *n* is given after the SD for each value. DBP, diastolic blood pressure; *E/e′*, ratio of peak transmitral flow velocity to peak mitral annular motion in early diastole; HR, heart rate; HFrEF, heart failure with reduced ejection fraction; LVEDD, left ventricular end-diastolic diameter; LVEF, left ventricular ejection fraction; SBP, systolic blood pressure. Where scans of both left and right carotids were obtained, heart rate for each patient was the mean of the two values.

### Wave Intensities

An example of corresponding diameter, velocity, and wave intensity waveforms is shown in Supplemental Fig. S3.

#### Intercycle reliability.

Wave intensities were relatively consistent between cardiac cycles. For a small number of participants, however, the breathing cycle produced appreciable variation in the diameter trace. Although velocity was generally more consistent, there were also participants for whom volatile heart rhythm and performance caused differences between cycles. It is for this reason that three scans were taken of each artery, and only one was selected for final analysis. If the scans achieved similar quality, the one with the highest number of cardiac periods in the ensemble was chosen.

#### Test-retest reliability.

Test-retest reliability was assessed for all metrics by calculating intraclass correlation coefficients (Supplemental Table S1). They were calculated for a larger group of participants than the wave metrics since exclusion criteria concerning age and echocardiogram availability were not relevant and were therefore dropped. Instead, participants were included if at least three measurements of wave intensity of satisfactory quality were obtained in a vessel.

Intraclass correlations exceeded or, in three cases only, approached 0.9 for SWI, SWE, S-D interval, ECG-S interval, and SWS, with the ECG-S interval in the right carotid reaching 0.99. D-wave and R-wave intensities and energies also showed good reliability, with coefficients ≥0.7 in all cases but two. The reflection index was the least reliable but still had a minimum coefficient of 0.55.

#### Wave metrics in two participant groups.

Supplemental Fig. S5 shows wave intensities as a function of time throughout the cardiac cycle for all individuals in the control and HFrEF groups for whom ECG and pulse wave measurements were synchronized. Control participants tended to have earlier, larger S waves, although there were clear exceptions.

**Figure 1.**
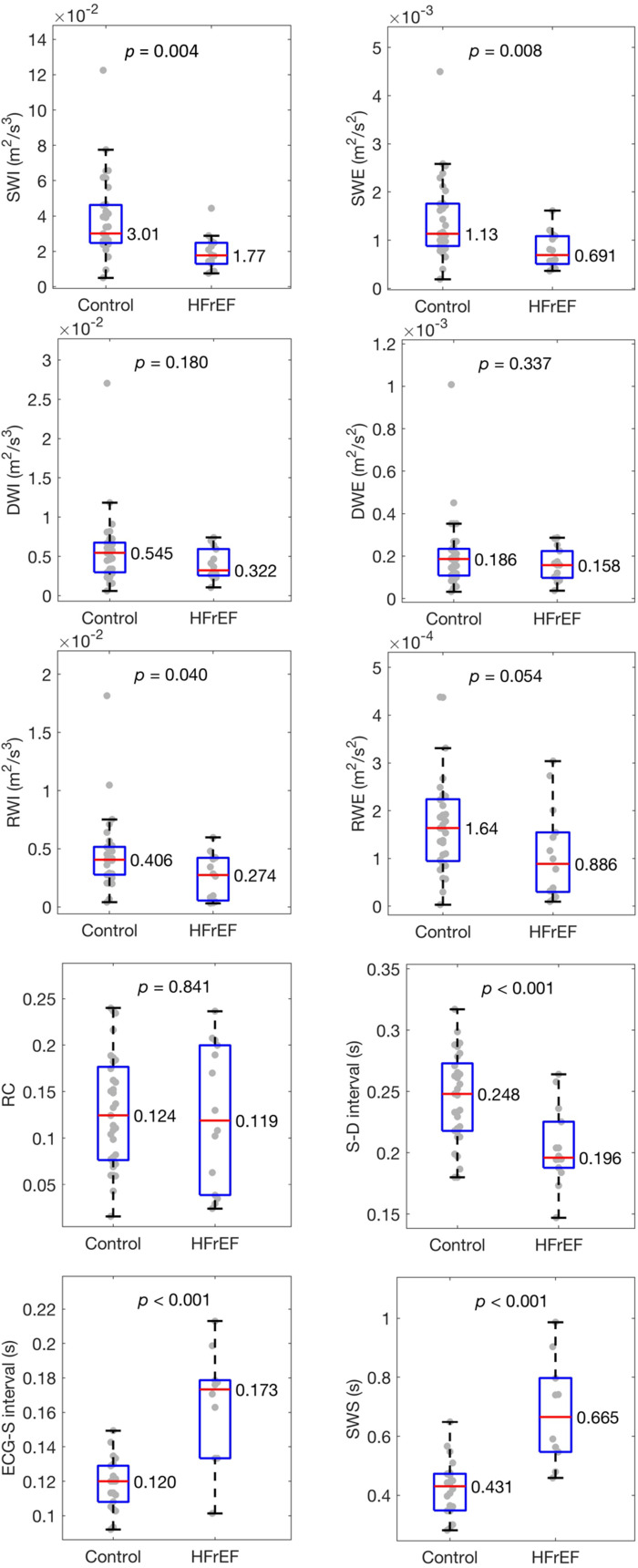
Wave metrics for the left carotid. Box and whiskers show minimum and maximum values, lower and upper quartiles, and medians. SWI, RWI, and DWI are intensities of the S, R, and D waves, respectively; SWE, RWE, and DWE are the corresponding energies; RC is the reflection index (or reflection coefficient); S-D and ECG-S intervals are the time between the arrival of the peaks of the S and D waves and between the peak of the R wave of the ECG and the arterial S wave, respectively; and SWS is S-wave shift; *n* = 33 control and 14 participants with HFrEF, respectively, except for ECG-S interval and SWS where *n* = 22 and 10. HFrEF, heart failure with reduced ejection fraction.

Boxplots showing the minimum and maximum, lower and upper quartiles, and median of each wave metric are given for the left and right carotids in Fig. 1 and Supplemental Fig. S6, respectively. Corresponding means, standard deviations, and *P* values are given in Supplemental Table S2. The SWI and SWE were lower, the S-D interval shorter, and the ECG-S interval and SWS longer in participants with HFrEF. All these differences reached significance in both vessels. RWI was also significantly lower, but RWE was not.

**Figure 2.**
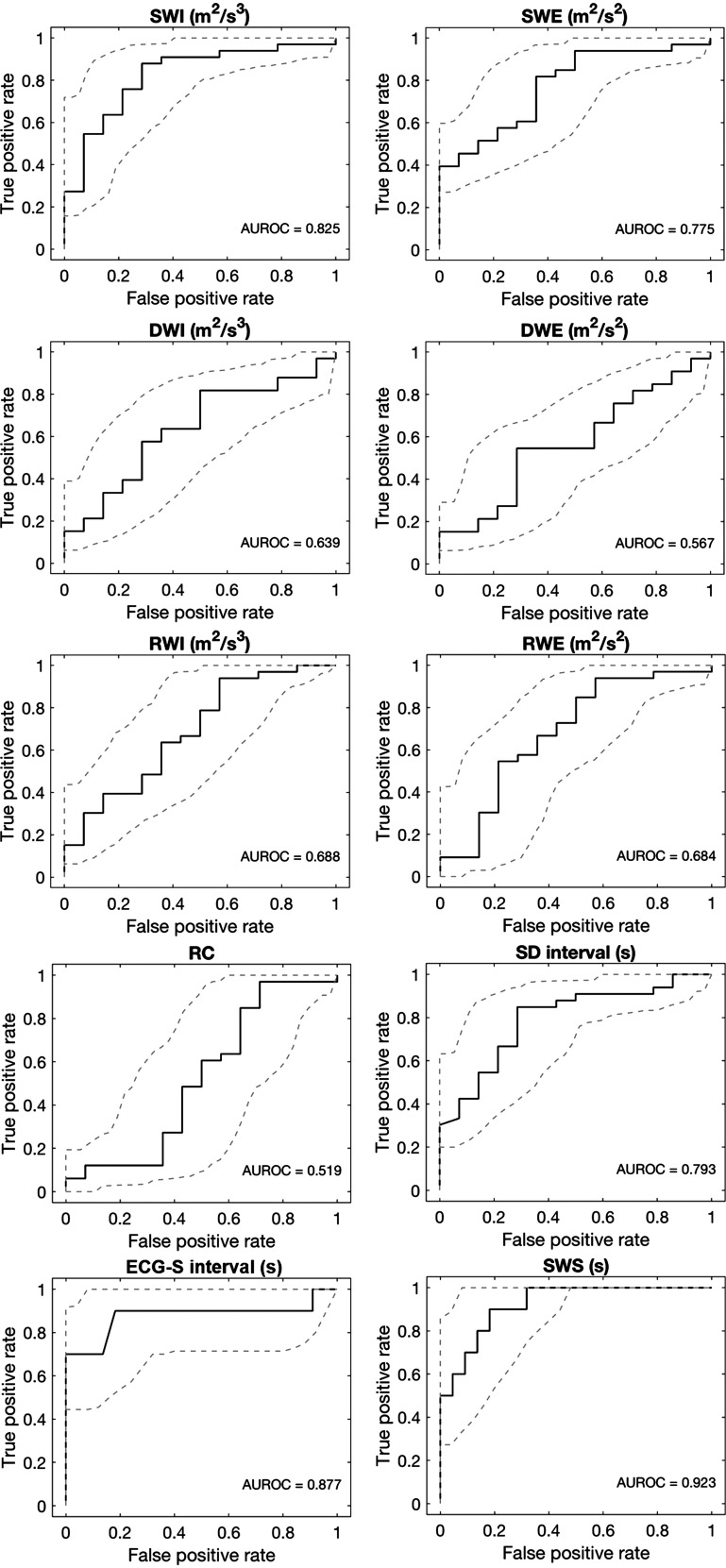
Identification of heart failure using left carotid wave metrics. The performance for each metric is expressed by a receiver operating characteristic (ROC). The area under the ROC (AUROC) is given inside the box for each metric. Dashed line: 95% confidence interval. SWI, RWI, and DWI are intensities of the S, R, and D waves; SWE, RWE, and DWE are the corresponding energies; RC is the reflection index (or reflection coefficient); the S-D and ECG-S intervals are the time between the arrival of the peaks of the S and D waves and between the peak of the R wave of the ECG and the arterial S wave; SWS is S-wave shift; *n* = 33 controls and 14 participants with HFrEF, except for ECG-S interval and SWS where *n* = 22 and 10. HFrEF, heart failure with reduced ejection fraction.

Receiver operating characteristics (ROCs) were used to describe the fraction of true positives (sensitivity) and the fraction of false positives (1 − specificity) obtained for each wave metric when examining the ability of different threshold values to determine whether individual participants had HFrEF or not. An area under the ROC (AUROC) of 0.7–0.8 is considered acceptable, 0.8–0.9 is considered excellent, and more than 0.9 is considered outstanding (25). The ROC and AUROC for each metric, together with the 95% confidence interval, are shown for the left and right carotids in Fig. 2 and Supplemental Fig. S7, respectively.

The D-wave intensities and energies gave AUROC values of 0.50–0.64, similar to the 0.5 expected for a random classifier. The S-wave intensities and energies gave higher values (0.76–0.83), and values for the reflection of the S wave were in between (0.68–0.73). The metrics based on timing gave high values: 0.69 and 0.79 for the S-D interval and 0.85 and 0.88 for the ECG-S interval in the right and left carotids, respectively. The values for SWS were even higher, at 0.88 and 0.92.

Note that AUROC values for SWI were broadly similar to those for SWE and the same is true for DWI and DWE and for RWI and RWE, as well as for the right and left carotids, giving additional confidence in the results.

**Figure 3.**
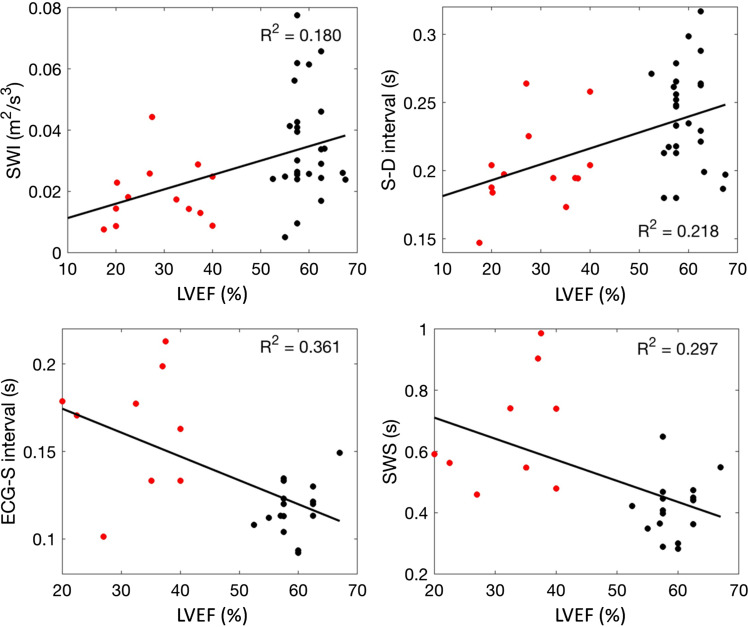
Participant-by-participant comparison of left carotid wave metrics with left ventricular ejection fraction. Participant ground truth classification: black, control; red, HFrEF. SWI is the intensity of the S wave; the S-D and ECG-S intervals are the time between the arrival of the peaks of the S and D waves and between the peak of the R wave of the ECG wave and the arterial S wave, respectively; SWS is the S-wave shift; *n* = 27 controls and 13 participants with HFrEF for SWI and S-D interval; *n* = 22 and 10 for ECG-S interval and SWS. HFrEF, heart failure with reduced ejection fraction.

Figure 3 and Supplemental Fig. S8 show scatter plots for the left and right carotids, respectively, in which SWI, S-D interval, ECG-S interval, and SWS are each compared with LVEF on a participant-by-participant basis for the subset of participants in which an ejection fraction was quantified. (Where LVEF was determined as a range, the middle value was used.) It is again evident that SWI and the S-D interval were on average higher in control participants than in participants with heart failure and that the ECG-S interval and SWS showed the opposite trend. It is also apparent that there was less overlap between the two groups for the ECG-S interval and SWS than for SWI and the S-D interval.

## DISCUSSION

Wave intensities were calculated from changes in arterial diameter and blood flow velocity, both measured noninvasively at the same location and time by tracking blood and wall speckles in a single sequence of B-mode images. This method is simple in principle, but there are two significant technical hurdles. The first is visualizing blood, which is a poor scatterer of ultrasound at the frequencies required to penetrate to the depth of large conduit arteries. That was solved by using singular value decomposition (SVD) to separate blood signals from wall signals and clutter. The second hurdle is the high frame rate required to capture overlapping patterns of blood speckles in successive frames. A frame repetition rate of 750–1,000 FPS was achieved by using an ultrafast scanner and plane-wave imaging.

The technique has been validated in preclinical studies (15). It gave patterns of wave intensity in the rabbit aorta that were consistent with patterns obtained using the original wave intensity formulation (*Eq. 1*) and an intra-arterial catheter to measure pressure and velocity. Furthermore, the technique detected reductions in SWI, SWE, and DWE comparable with those obtained with the intra-arterial catheter when ventricular dysfunction was induced by a cardioselective β-blocker. The technique also gave patterns of wave intensity consistent with the studies of Curtis et al. (2) and physiologically realistic wave speeds in the carotid artery of a healthy human participant.

Here we applied the technique to HFrEF and control participants to determine whether it could detect the expected differences in their wave characteristics. Participants with HFrEF had a significantly lower SWI, SWE, and RWI, a shorter S-D interval, and a longer ECG-S interval and SWS in both carotids. For the right carotid, Curtis et al. (2) observed changes in SWE, S-D interval, and ECG-S interval of −37, −15, and +25%, respectively. We obtained −34, −12, and +33% for the equivalent metrics in the same vessel. The first two figures are slightly smaller in our study, but the percent change in the ECG-S interval was larger. Furthermore, coefficients of variation for the ECG-S delay in our study were on average less than half of those in the Curtis et al. study. [Coefficients of variation in Curtis et al. (2), Sugawara et al. (3), and the present study are given in Supplemental Table S3.] The discrepancy may reflect the nonsimultaneous acquisition of P and *U* in the study by Curtis et al. (2) or the use of *D* rather than *U* to compute wave intensity in the present study.

We did not observe significant differences between groups for DWI or DWE. Curtis et al. (2) and Sugawara et al. (3) also failed to find a significant difference in patients with systolic dysfunction.

The changes are consistent with participants with HFrEF having had weaker, shorter, and delayed ventricular ejection. The velocity of contraction is known to be reduced in systolic heart failure (26), and that may in turn reflect the well-established impairment of myocyte Ca^2+^ handling. More specifically, there are smaller transients in cytoplasmic Ca^2+^ concentration during the cardiac cycle, and hence reduced contractile function (27). Alterations of proteins involved in Ca^2+^ handling occur early in heart failure and may precede alteration of pump function (28).

Test-retest reliability was high in general and particularly so for SWI, S-D interval, ECG-S interval, and SWS. However, clinical utility requires more than high reliability and the ability to detect significant differences between groups of participants; the technique must also have sufficient sensitivity and specificity that it can be used to determine whether each patient has or does not have heart failure. That has not been addressed in previous studies of altered wave intensity and was quantified here by the area under the receiver operating characteristic (AUROC).

Circulating concentrations of natriuretic peptides are an established diagnostic tool. In a study by Zaphiriou et al. (29) involving 306 patients with suspected heart failure, referred by their general practitioners, the AUROC was 0.84 for brain natriuretic peptide (BNP) and 0.85 for NH_2_-terminal pro-BNP (NTproBNP). The present study used a smaller sample and focused on HFrEF. It obtained lower AUROC values for SWI, SWE, and the S-D interval, but an approximately equal value for the ECG-S interval. The compound metric SWS gave values of up to 0.92.

The SWS metric has conceptual similarities with the preejection period (PEP) to left ventricular ejection time (LVET) ratio (30), although it is based on the timing of waves rather than valve opening and closing. When calculating it, the S-D interval was normalized by heart period, whereas the ECG-S was not, because our data showed a nearly direct proportionality between the S-D interval and heart period but no relation between the ECG-S interval and heart period (Supplemental Fig. S9).

Preliminary exploration of a Logistic Regression Model using as inputs the ECG-S interval, S-D interval, and heart rate, the three variables used to compute SWS, raised the left carotid AUROC to 0.96, and perfect discrimination of the 32 controls and participants with HFrEF (i.e., AUROC = 1) was achieved by adding the SWI.

Finally, we consider the unmet clinical need for heart failure screening in primary care. Between 2010 and 2013 in the United Kingdom, 79% of patients with heart failure were diagnosed after acute admission to hospital, even though 41% had presented to their general practitioners with relevant symptoms in the preceding 5 years (31). The BNP test has a poor take-up in many geographic areas, despite its recommendation by the National Institute for Health and Care Excellence (32). Much current research is aimed at developing new methods for HF screening by nonspecialists ((33). The present study suggests that a focus on the timing of the S wave may answer this need. However, that remains a hypothesis: the LVEF of participants was defined by echocardiography, and consequently, the control and HFrEF groups had to be recruited from cardiology clinics, which may have led to a profile of conditions and treatments that is unrepresentative of primary care. (Supplemental Table S4 lists medication for the two groups of participants.)

Minimal training is required to operate the equipment used here and to interpret the output, which is not the case with echocardiography. However, the methods are based on sophisticated apparatus and algorithms. Cheaper scanners and computers would be desirable. One avenue for exploration is the use of decorrelation methods for assessing fluid velocity (34) as they require fewer ultrasound lines and avoid the computationally demanding need to obtain cross correlations for groups of speckles across an entire image. Another is the use of sparse sampling (35).

## DATA AVAILABILITY

Data will be made available upon reasonable request.

## SUPPLEMENTAL DATA

Supplemental Figs. S1–S9 and Supplemental Tables S1–S4: http://hdl.handle.net/10044/1/111286.

## GRANTS

This work was supported by British Heart Foundation Grant PG/18/48/33832 (to P.D.W., J.M., and M.-X.T.). R.M.R. was supported by a Studentship from the Engineering and Physical Sciences Research Council Centre for Doctoral Training in Fluid Dynamics Across Scales. A.R. was supported by a National Institutes of Health Fellowship.

## DISCLOSURES

P.D.W. is named as inventor on patents filed by or granted to Imperial College London that describe some of the underlying technology. None of the other authors has any conflicts of interest, financial or otherwise, to disclose.

## AUTHOR CONTRIBUTIONS

R.M.R., A.R., E.M.R., M.-X.T., J.M., and P.D.W. conceived and designed research; R.M.R., A.R., and E.M.R. performed experiments; R.M.R., A.R., E.M.R., and P.D.W. analyzed data; R.M.R., A.R., E.M.R., M.-X.T., J.M., and P.D.W. interpreted results of experiments; R.M.R., A.R., E.M.R., and P.D.W. prepared figures; R.M.R., A.R., E.M.R., and P.D.W. drafted manuscript; R.M.R., A.R., E.M.R., M.-X.T., J.M., and P.D.W. edited and revised manuscript; R.M.R., A.R., E.M.R., M.-X.T., J.M., and P.D.W. approved final version of manuscript.
